# The High Temperature Tensile and Creep Behaviors of High Entropy Superalloy

**DOI:** 10.1038/s41598-017-13026-7

**Published:** 2017-10-04

**Authors:** Te-Kang Tsao, An-Chou Yeh, Chen-Ming Kuo, Koji Kakehi, Hideyuki Murakami, Jien-Wei Yeh, Sheng-Rui Jian

**Affiliations:** 1 Department of Materials Science and Engineering, National Tsing Hua University, Hsinchu, 30013 Taiwan R.O.C.; 2 Department of Mechanical and Automation Engineering, I-Shou University, Kaohsiung, 84001 Taiwan R.O.C.; 3 Department of Mechanical Engineering, Tokyo Metropolitan University, 1-1 Minami-osawa, Hachioji-shi, Tokyo, 192-0397 Japan; 4 National Institute for Materials Science, Sengen 1-2-1, Tsukuba, Ibaraki, 305-0047 Japan; 5 Department of Materials Science and Engineering, I-Shou University, Kaohsiung, 84001 Taiwan R.O.C.

## Abstract

This article presents the high temperature tensile and creep behaviors of a novel high entropy alloy (HEA). The microstructure of this HEA resembles that of advanced superalloys with a high entropy FCC matrix and L1_2_ ordered precipitates, so it is also named as “high entropy superalloy (HESA)”. The tensile yield strengths of HESA surpass those of the reported HEAs from room temperature to elevated temperatures; furthermore, its creep resistance at 982 °C can be compared to those of some Ni-based superalloys. Analysis on experimental results indicate that HESA could be strengthened by the low stacking-fault energy of the matrix, high anti-phase boundary energy of the strengthening precipitate, and thermally stable microstructure. Positive misfit between FCC matrix and precipitate has yielded parallel raft microstructure during creep at 982 °C, and the creep curves of HESA were dominated by tertiary creep behavior. To the best of authors’ knowledge, this article is the first to present the elevated temperature tensile creep study on full scale specimens of a high entropy alloy, and the potential of HESA for high temperature structural application is discussed.

## Introduction

The concept of high entropy alloys (HEAs) has allowed the exploration of large composition space of alloys, and is currently one of the most intriguing research topics in the field of materials science^1–3^. Recent studies have shown attractive mechanical properties of HEAs at cryogenic temperature^4^ and room temperature^5^ with excellent combinations of high strength and toughness, indicating their potential as the structural materials. However, the strength of single phase type HEAs were reported to be insufficient at elevated temperatures^6^. Several HEAs, such as CoCrFeNiNb_x_^7^ and CoCrFeNiMo_x_^8^ demonstrated that a moderate increase in tensile strength can be achieved by various precipitates. As a result, precipitation strengthening should be considered in developing HEAs for high temperature applications. The physical metallurgy of superalloys^9^ have been employed to design HEAs by utilization of significant volume fractions of stable and coherent L1_2_ γ′ to provide high temperature strength, e.g., Al_10_Co_25_Cr_8_Fe_15_Ni_36_Ti_6_ HEA was designed to contain 46 vol% of γ′ precipitates^10^, and its tensile strength could surpass those of Inconel 617 and Alloy 800 H. Nevertheless, the γ′ solvus temperatures of the reported γ′-bearing HEAs still remained relatively low comparing to those of superalloys^9^. Furthermore, there were phase instability associated with the formation of L2_1_ Ni_2_AlTi, B2, and Cu-rich FCC^5,11,12^. Therefore, the development for precipitation strengthened HEAs is only at the incipient stage, and more research efforts will be required.

In present study, the high temperature tensile and creep behaviors of a novel alloy named high entropy superalloy (HESA)^13^ have been investigated. HESA is strengthened by the coherent L1_2_ γ′ precipitates, while the FCC matrix remains high entropy (ΔS_mix_ > 1.5 R)^14^. For example, the composition of HESA in current study is Ni_47.9_Al_10.2_Co_16.9_Cr_7.4_Fe_8.9_Ti_5.8_Mo_0.9_Nb_1.2_W_0.4_C_0.4_ (at%), so the calculated ΔS_mix_ is 1.60 R, which is higher than those of conventional superalloys such as CM247LC (ΔS_mix_ = 1.29 R), Rene′ N5 (ΔS_mix_ = 1.22 R), and CMSX-2 (ΔS_mix_ = 1.14 R). Previous studies have shown that the γ - γ′ microstructure of HESAs can remain stable against topologically-close-packed (TCP) phase formation from 700 to 1100 °C for at least 500 h^15^, and with enhanced γ′ solvus temperatures above 1150 °C^16^. Furthermore, HESAs have shown good resistances against high temperature oxidation and corrosion by forming protective Al_2_O_3_ and Cr_2_O_3_, respectively^17^. However, the tensile and creep behaviors of HESA have not yet been reported. Therefore, these two properties are the main subject of focus in this article. To the best of authors’ knowledge, there is no reported creep studies on the full scale sample of HEAs at elevated temperature such as 982 °C. Although some studies utilized nano-indentation method to deduce creep mechanism of HEA^18,19^, these tests could not take the effect of long-term microstructure stability on creep into consideration. Therefore, this article presents the first tensile creep studies on full size specimen of a high entropy alloy, which can benefit the community of materials science and engineering whose interest is in the development of HEAs for elevated temperature applications.

## Results

**Figure 1:**
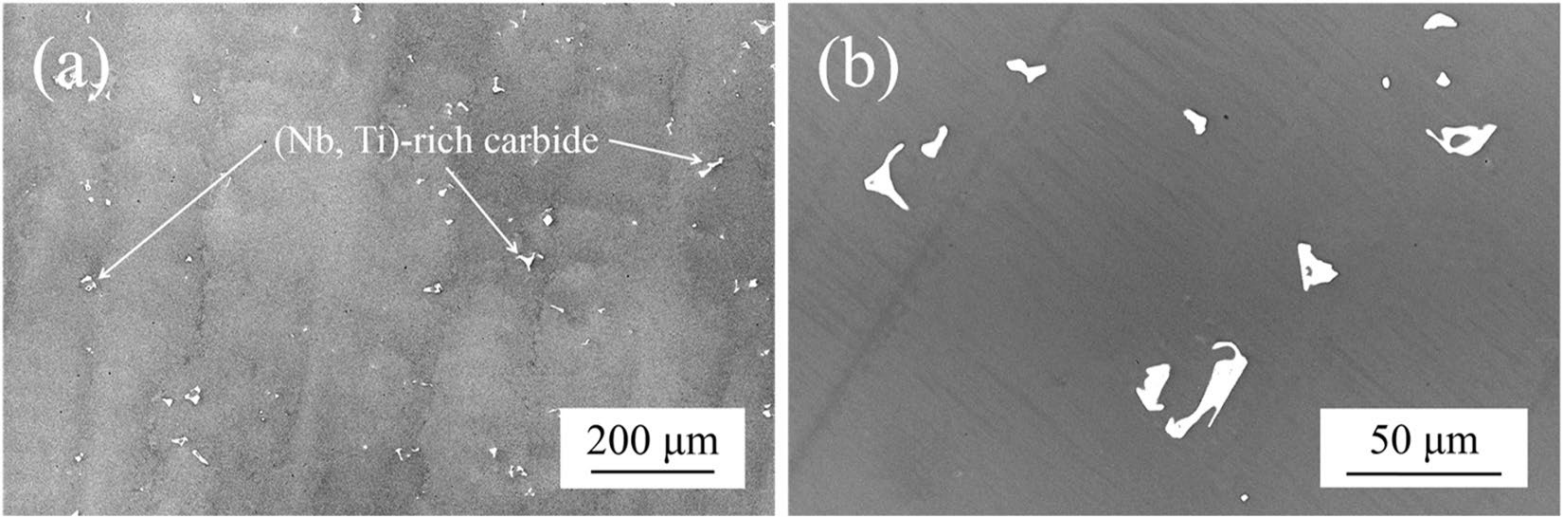
The back-scattered electron images of the **(a)** directionally-solidified dendritic structure of as-cast HESA with **(b)** the presence of carbides.

**Figure 2:**
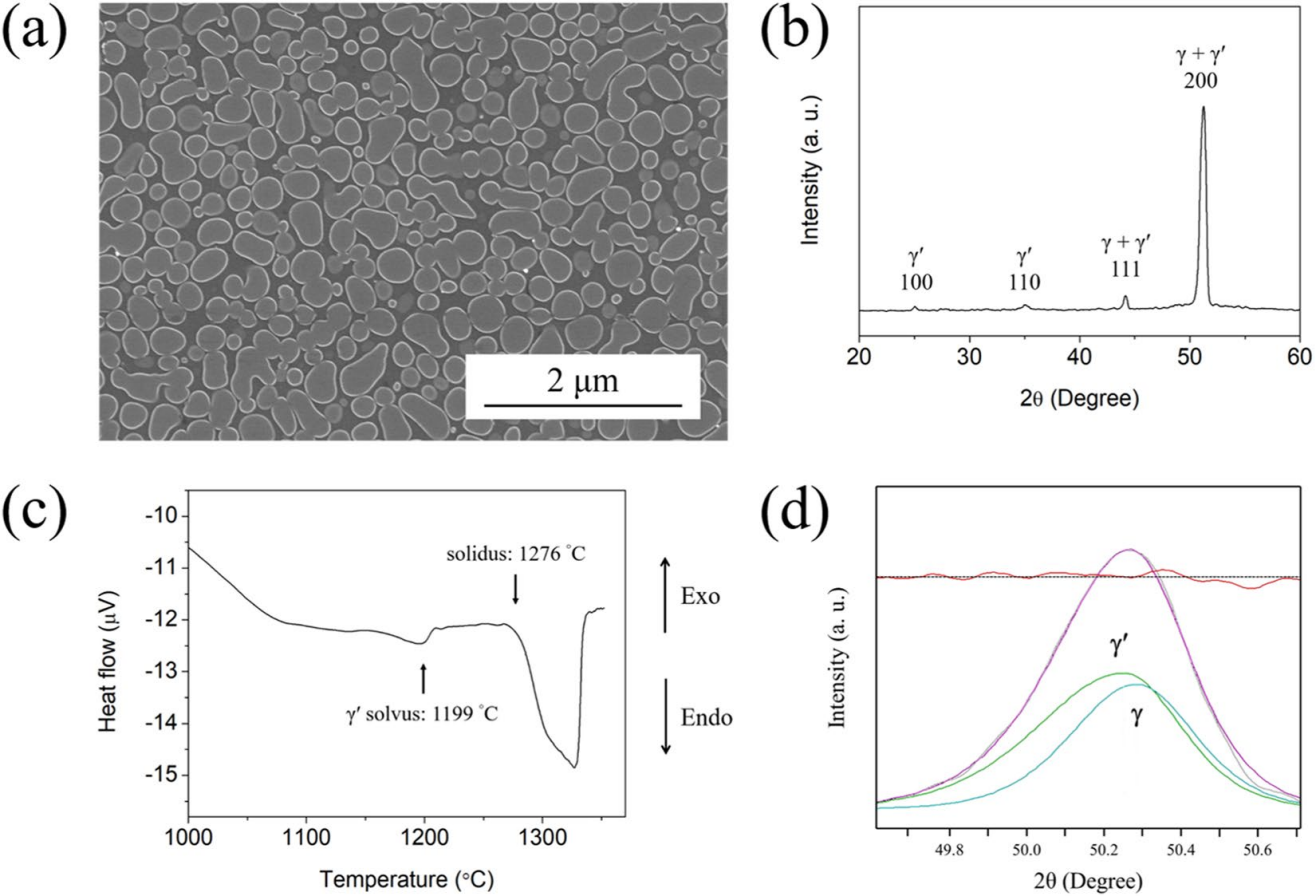
(**a**) The typical γ/γ′ microstructure of HESA and **(b)** the XRD characteristic peaks of γ and γ′ phases. **(c)** The DTA curve during heating after solution and aging heat treatments. **(d)** Deconvolution of {200} reflection of HESA at 982 °C.

Figure 1(a) shows the SEM back-scattered electron image of the as-cast HESA, which consists of dendritic microstructure with some carbides. The morphology of carbide is shown in Fig. 1(b), and the average chemical composition measured by SEM-EDS suggests that these are (Nb, Ti)-rich MC carbides, Table 1. After the solution and aging heat-treatments, the microstructure consists of γ matrix and dispersed γ′ precipitates is shown in Fig. 2(a). The γ′ precipitates appears to be spherical with an average size of 290 nm and 69 vol% measured by image analysis. Figure 2(b) shows the XRD scan at room temperature, and the characteristic peaks confirm the FCC + L1_2_ phases of HESA. The DTA curve during heating is shown in Fig. 2(c). It also indicates the two phases microstructure, and the γ′ solvus and solidus temperature are 1199 and 1276 °C, respectively. In addition, deconvolution of {200} reflection of XRD scan at 982 °C allows the measurement of lattice misfit at high temperature, Fig. 2(d); peak intensity ratio between γ and γ′ can be correlated with their relative volume fractions for determining lattice constants of γ and γ′ to be 3.625 and 3.628 Å, respectively. Therefore, by the calculation of $\delta =2({a}_{{\rm{\gamma }}^{\prime} }-\,{a}_{\gamma })/({a}_{{\rm{\gamma }}^{\prime} }+{a}_{\gamma })$, where *δ* is the lattice misfit between γ and γ′, *a*_*γ*_ and ${a}_{{\gamma }^{^{\prime} }}$ is the lattice constant of γ and γ′ phase, respectively, the lattice misfit of HESA at 982 °C is 0.08%.

**Table 1:** The nominal composition of HESA and CMSX-2^21^, and the measured compositions of γ, γ′ and carbide in these alloys.

at%	Ni	Al	Co	Cr	Fe	Ti	Mo	Ta	Nb	W	C
**HESA**											
nominal	47.9	10.2	16.9	7.4	8.9	5.8	0.9	—	1.2	0.4	0.4
γ	41.0	6.8	21.8	11.9	12.9	2.8	1.3	—	1.0	0.5	—
γ′	52.5	11.4	13.1	5.1	7.4	8.2	0.3	—	1.6	0.4	—
carbide	—	—	—	—	—	17.1	2.0	—	33.1	3.3	44.5
**CMSX-2**											
nominal	67.4	12.5	5.0	9.0	—	1.2	0.4	2.0	—	2.5	—
γ	59.0	3.1	8.6	25.5	—	0.6	0.6	0.1	—	2.5	—
γ′	70.5	16.7	3.2	2.4	—	1.6	0.2	3.0	—	2.4	—

**Figure 3:**
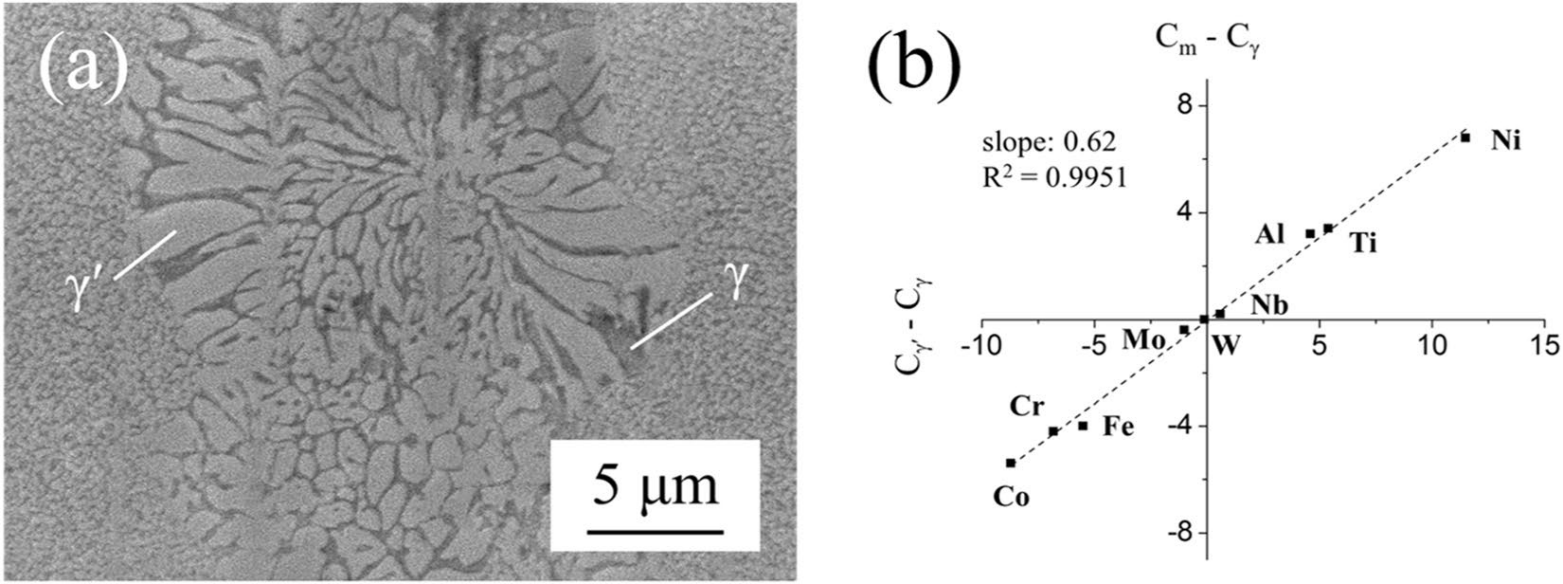
(**a**) The strain-aged cellular microstructure of γ/γ′, and **(b)** plot of ${{\rm{C}}}_{\gamma {\rm{^{\prime} }}}$ - C_γ_ vs. C_m_ - C_γ_ of HESA.

The chemical composition of γ and γ′ phase has been determined by strain-aging method^20^ in this study. Figure 3(a) shows the cellular microstructure of γ and γ′ phase after strain-aging, and the average compositions of individual γ and γ′ measured by SEM-EDS are summarized in Table 1 along with those of a conventional superalloy CMSX-2 reported in literature^21^, which contains an average 68 vol% of cuboidal shape γ′ precipitates. By utilizing the chemical compositions, Fig. 3(b) can be plotted with ${{\rm{C}}}_{\gamma {\rm{^{\prime} }}}$ - C_γ_ vs. C_m_ - C_γ_, where C_m_ is the nominal composition, ${{\rm{C}}}_{\gamma {\rm{^{\prime} }}}$ is the γ′ composition, and C_γ_ is the γ composition. The linear fitting slope in Fig. 3(b) is 0.62, which is close to the γ′ volume fractions measured experimentally, so the validity of the measured γ and γ′ compositions has been demonstrated. According to Table 1, there are relatively high Co and Ti concentrations in both γ and γ′ phases of HESA. Since Co and Ti additions can reduce the stacking fault energy (SFE) of a Ni matrix^22^, lower SFE of γ matrix in HESA can be expected, as the generally reported low SFEs of HEAs^23,24^. A functional model has been utilized to estimate the SFE of HESA^25^. The fractional change of γ SFE due to alloying can be described as: ${{\rm{\Delta }}}_{\Gamma }^{\gamma }(x)=\sum _{i}{x}_{i}\delta {\Gamma }_{i}$, where *Γ* is the stacking fault energy and $\delta {\Gamma }_{i}$ is the optimized model parameter value for the effect of alloying element on SFE of pure Ni, Table 2 ^25^. By applying the γ compositions in Table 1 into the above model, the value of ${{\rm{\Delta }}}_{\Gamma }^{\gamma }(x)$ are −1.0 and −0.67 for HESA and CMSX-2, respectively, indicating that the reduction of SFE in the Ni-based FCC matrix due to alloying in HESA can be 1.5 times greater than that of CMSX-2. According to references, the SFE of pure Ni is 1.3 J/m^2^ and that of CMSX-2 is 0.9 J/m^2^ ^26^, so the reduction of SFE due to alloying for CMSX-2 is 0.4 J/m^2^, while that for HESA should be 0.6 J/m^2^. Therefore, the SFE of the HEA matrix can be estimated around 0.7 J/m^2^. Furthermore, high Ti content in γ′ phase can significantly increase the anti-phase boundary (APB) energy^27^. According to JMatPro calculations, the APB energies of γ′ phase in HESA and CMSX-2 are 0.22 and 0.19 J/m^2^, respectively. Consequently, higher APB energy of γ′ may contribute to some degree of strengthening in HESA^28^.

**Table 2:** The optimized model parameters (δΓ_i_) for the effect of alloying elements on stacking fault energy of pure Ni^25^.

	Ni	Al	Co	Cr	Fe
δΓ_i_	—	−1.2362	−0.6240	−1.6964	−3.0378
	**Ti**	**Mo**	**Ta**	**Nb**	**W**
δΓ_i_	−2.8888	−4.0986	−3.5682	−3.9847	−3.8707

**Figure 4:**
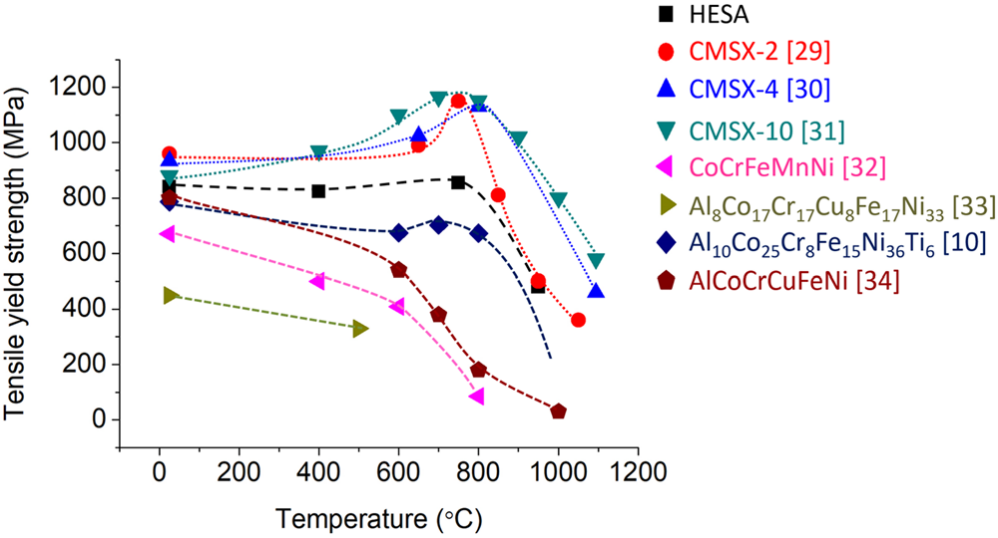
The tensile yield strength of HESA, Ni-based superalloys^29–31^ and high entropy alloys^10,32–34^ from room to high temperature.

The tensile yield strength versus temperature plot of HESA comparing to those of the commercial Ni-based superalloys^29–31^ and reported high entropy alloys^10,32–34^ are shown in Fig. 4. The precipitation strengthening has allowed HESA to possess higher tensile yield strengths than those of CoCrFeMnNi throughout the temperatures. Furthermore, the tensile yield strengths of HESA are the highest among reported high entropy alloys. Although the strengths of HESA are lower than those of CMSX-4 and CMSX-10, its behavior above 800 °C can be close to those of CMSX-2.

**Figure 5:**
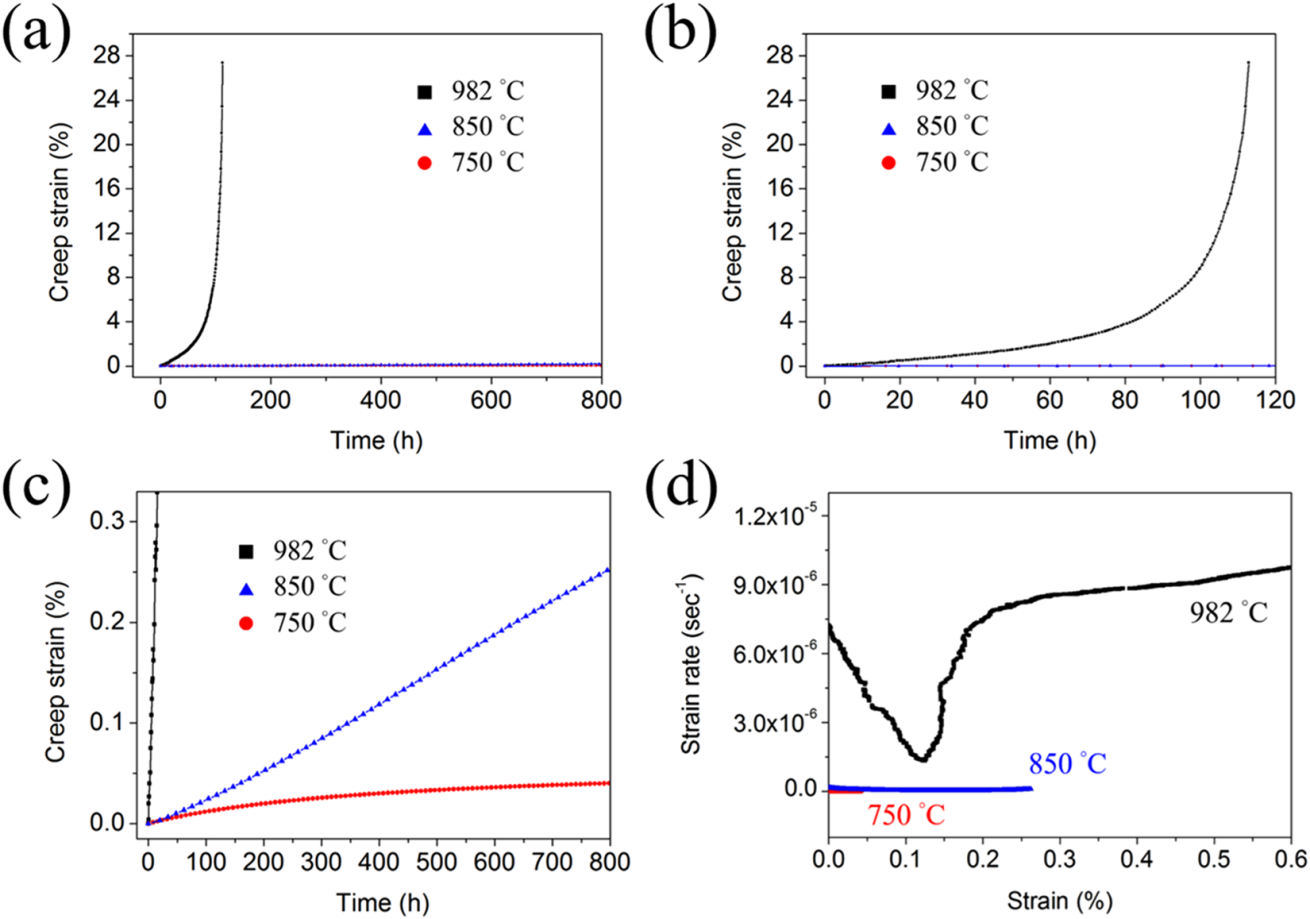
(**a**) The 982, 850 and 750 °C/159 MPa creep curves, and the enlarged fragment of **(a)** within **(b)** 120 h and **(c)** 0.33% strain. **(d)** The creep strain rate vs. strain curves of HESA.

**Figure 6:**
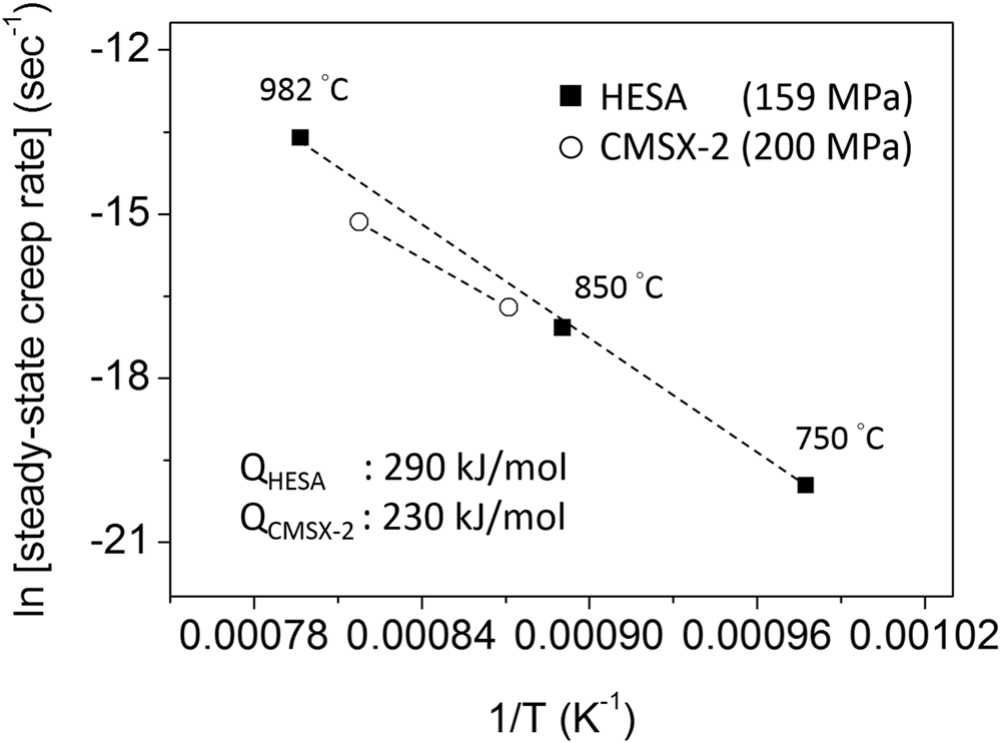
The steady-state creep rate vs. T^−1^ plot of HESA and CMSX-2^37^.

Creep behaviors of HESA at 982, 850 and 750 °C under 159 MPa are shown in Fig. 5a,b,c. The creep rupture life of HESA at 982 °C is about 114 h, which is similar to those of several first-generation Ni-based superalloys under similar test conditions, e.g., the creep life of NX-188 DS under 982 °C/138 MPa test is 58 h^35^, and that of Rene′ 80 under 982 °C/145 MPa test is 118 h^36^. The 982 °C creep curve of HESA is clearly shown in Fig. 5(b), and there is no obvious primary creep with gradual increase in creep strain rate corresponding to an extensive tertiary creep behavior. Figure 5(c) enlarges the creep strain axis of 750 and 850 °C creep curves, and there are still no obvious primary creep regime. After 800 h interrupted tests, the creep strains only have accumulated to 0.04% and 0.25% at 750 °C and 850 °C, respectively. The creep strain rates versus strain curves are shown in Fig. 5(d), and by plotting the minimum creep strain rate versus T^−1^, where T is the temperature, the dependence of creep strain rate on the testing temperature can be shown in Fig. 6; the creep strain rates of CMSX-2 under 200 MPa load are also included in this figure (5.6 × 10^−8^ sec^−1^ at 875 °C and 2.6 × 10^−7^ sec^−1^ at 948 °C^37^). Dependence of the steady-state creep rate ($\dot{{\rm{\varepsilon }}}$) on the applied stress (σ) and temperature (T) can be expressed as: $\dot{\varepsilon }={\rm{A}}{\sigma }^{n}\exp (-{\rm{Q}}/{\rm{RT}})$, where A is a constant, Q is the activation energy for creep and R is the gas constant^9^, thus the activation energy for creep (Q) can be further described as: ${\rm{Q}}=-{\rm{R}}(-\partial \,\mathrm{ln}\,\dot{\varepsilon }/\partial {T}^{-1})$ under constant loads. From the linear slope of $\mathrm{ln}\,\dot{{\rm{\varepsilon }}}$ vs. T^−1^ curve, the creep activation energy of HESA can be estimated to be 290 kJ/mol, while that of CMSX-2 is 230 kJ/mol^37^.

**Figure 7:**
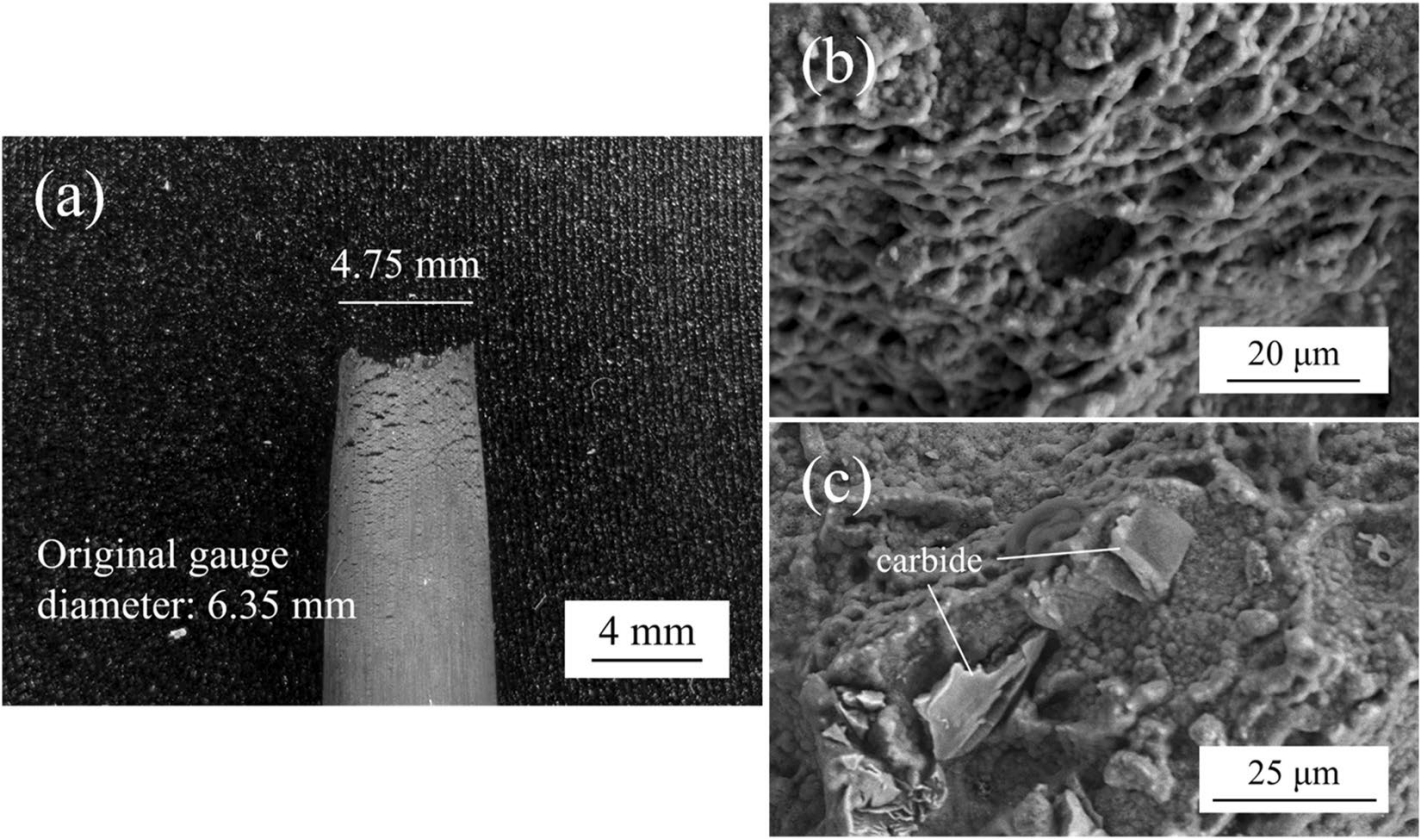
(**a**) The side view of fractured gauge, and the transverse fractograph of **(b)** dimple structure and **(c)** partially brittle fracture of the 982 °C/159 MPa as-crept HESA.

**Figure 8:**
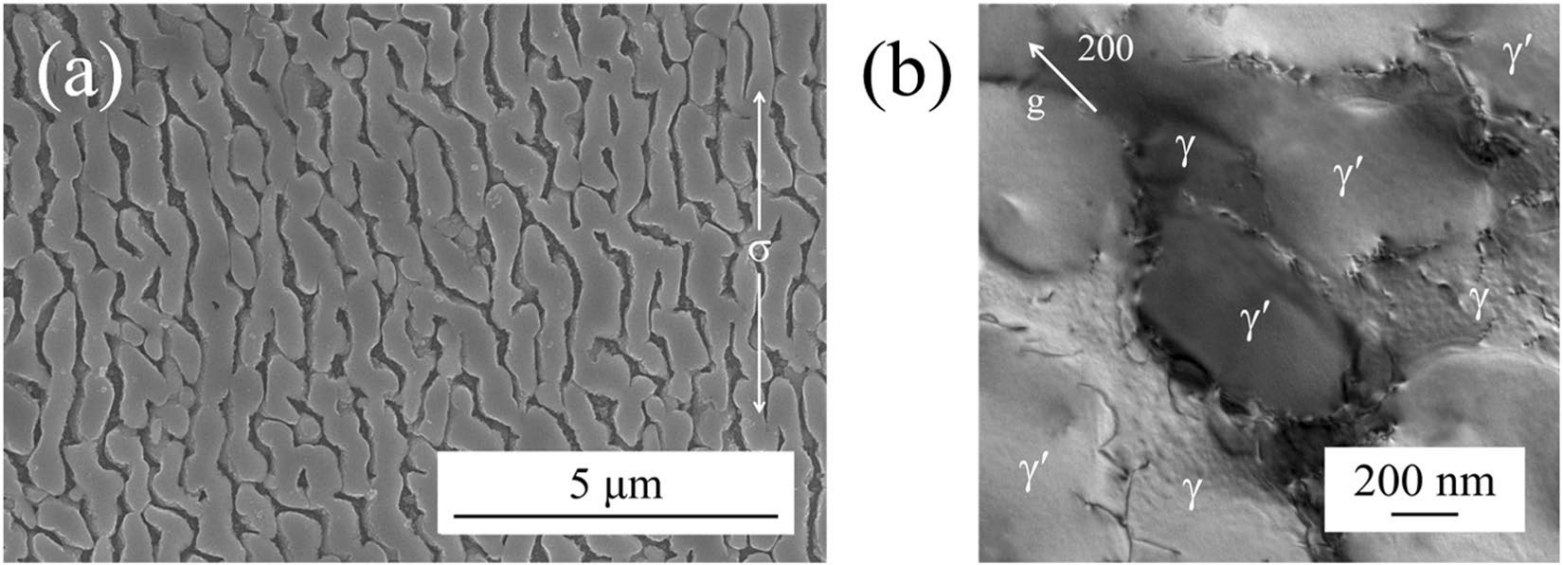
(**a**) The γ′ rafting parallel to the stress axis, and **(b)** the dislocation morphology of the 982 °C/159 MPa as-crept HESA.

**Figure 9:**
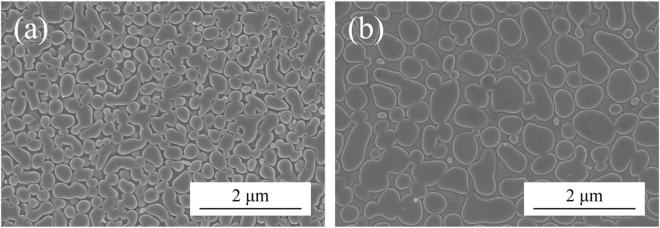
The un-rafted γ′ microstructure of HESA after 800 h creep tests at **(a)** 750 °C and **(b)** 850 °C.

Figure 7(a) shows the side view of the 982 °C crept rupture specimen of HESA. The diameter of the fractured gauge region has decreased from the original 6.35 mm to 4.75 mm, which demonstrates a ductile necking behavior before fracture. In addition, it appears to be no severe stress concentration occurred during creep deformation. The transverse fractograph are shown in Fig. 7b,c. According to Fig. 7(b), the fracture surface is rough and with the majority dimple feature corresponding to ductile behaviour; however, minor brittle fracture regions reflected by facet-like morphology can be observed associated with the presence of carbides, Fig. 7(c). Microstructure evolution during high temperature creep is known to influence creep behaviour, such as the directional coarsening of γ′ precipitates – “rafting”^38^. The plate-like γ′ morphology of the 982 °C crept HESA is shown in Fig. 8(a). The average diameter of γ′ perpendicular to the stress axis has coarsened from 0.29 μm to 0.5 μm, while the average length in parallel direction extended to 3 μm. Therefore, the rafting of γ′ in HESA has shown to coarsen along the direction of the applied stress, which can be related to the positive lattice misfit (δ) between γ and γ′^39^. Figure 8(b) shows the TEM analysis of crept HESA at 982 °C; dislocations have mainly piled-up within the γ matrix with no shearing the γ′ phase. Figure 9a,b show the microstructures of HESA after 800 h interrupted creep at 750 and 850 °C. The microstructure can be stable with high entropy γ matrix and L1_2_ γ′, while the size of γ′ has increased from 0.3 μm to 0.4 and 0.6 μm after 750 and 850 °C creep tests, respectively. Moreover, the morphology of γ′ has remained spherical, indicating that no rafting of γ′ has occurred in both conditions. These stable γ′ morphologies can be beneficial to resist creep deformation, and further discussions is presented in the next section.

## Discussion

The high temperature tensile and creep properties of a precipitation strengthened high entropy alloy, i.e., high entropy superalloy (HESA) have been investigated. Comparing to those of the reported high entropy alloys, HESA possesses outstanding tensile yield strength from ambient to elevated temperatures. Furthermore, the creep rupture life of HESA at 982 °C can be compared to those of Ni-based superalloys such as RENE′ 80 and NX-188 DS, so the potential of HESA for high temperature applications is demonstrated in this article.

The tensile strength of HESA can be attributed to the high volume fraction of strengthening precipitates γ′, while previous HEAs like Al_10_Co_25_Cr_8_Fe_15_Ni_36_Ti_6_ contains only 46 vol% of γ′ at room temperature^10^. As compared with the tensile yield strength of CMSX-2, the underlying reason of the slightly lower tensile yield strength can be elucidated by the following section. According to strong-pair and weak-pair coupling theories^40,41^, the critical resolved shear stress (τ_c_) of the precipitation strengthened alloys can be determined by the factors of APB energy, volume fraction of γ′ and γ′ size as: ${\tau }_{c}=\frac{{\gamma }_{APB}}{2b}[{(\frac{6{\gamma }_{APB}fr}{\pi T})}^{1/2}-f]$ and ${\tau }_{c}=\sqrt{\frac{3}{2}}(\frac{Gb}{r})\,{f}^{1/2}\frac{w}{{\pi }^{3/2}}{(\frac{2\pi {\gamma }_{APB}r}{wG{b}^{2}}-1)}^{1/2}$, where *γ*_*APB*_ is the APB energy, *f* is the volume fraction of γ′, *r* is the γ′ radius, *T* is the line tension, *G* is the shear modulus, *b* is the Burgers vector and *w* is a dimensionless constant. Since these theories have indicated that the peak strength would be yielded as the r increases to around $\frac{2T}{{\gamma }_{APB}}$ ^9^, and with the similar volume fraction of γ′ between HESA (69 vol%) and CMSX-2 (68 vol%), the optimized size of γ′ for peak strength in HESA can be estimated from that of CMSX-2. According to JMatPro calculations, the APB energy of HESA and CMSX-2 is 0.22 and 0.19 J/m^2^, respectively. In addition, due to the tensile strength of CMSX-2 has been reported to be significantly improved with an increase in average γ′ size from 300 nm to 450 nm^42^, and with the inversely proportional relationship to APB energy, the optimized size of γ′ in HESA should be around 390 nm; however, the as heat-treated γ′ size is only 290 nm in this work. As the τ_c_ is proportional to r^1/2^, the strength of HESA may increase by 16% when its γ′ size increases from 290 to 390 nm, which would contribute to the room temperature tensile yield strength increment from 840 MPa to 960 MPa. Furthermore, by comparing the increase in tensile yield strength from room temperature to the peak strength (Δσ_y_) of CMSX-2 (960 to 1150 MPa) and HESA (840 to 855 MPa), the magnitude of positive temperature dependence on yield strength of HESA appears to be less pronounced. For an alloy with coherent precipitates, the constrained lattice misfit (δ) can also affect the mechanical strength, i.e., higher degree of |δ| could lead to higher increase in yield strength due to the stress field around the precipitates interact with dislocations^43,44^. Since the δ of CMSX-2 was reported around −0.33%^45^, its higher degree of misfit value would contribute to the higher strength. From a designer point of view, the tensile yield strength of HESA appears to be very stable from room temperature to 800 °C, which could be more favorable than conventional superalloys for gas turbine engine application.

Regarding to creep behaviors, rafting can strongly affect the deformation at elevated temperatures. The coarsening of γ′ in CMSX-2 has been reported to be perpendicular to the stress axis (N-raft)^37^, so the N-rafted γ′ can provide interfaces with the formation of equilibrium interfacial dislocation network to hinder dislocation movements^46^. By contrast, the creep of HESA is dominated by parallel raft (P-raft) and without perpendicular γ/γ′ interfaces. Tetzlaff *et al*. have reported that tertiary creep may dominate the creep response of P-rafted superalloys^47^, and this is exactly the creep behavior observed for HESA at 982 °C. Therefore, higher creep rate of HESA compared to that of CMSX-2 could be a result of P-raft. According to a recent work on the thermal cycling creep of Ni-based superalloys, the P-raft of γ′ is proposed to be instead beneficial to the thermal cyclic creep resistance^48^. Consequently, the non-isothermal creep behavior of HESA may be worth studying in the future. On the other hand, HESA can exhibit the more comparable creep resistances to those of CMSX-2 at intermediate temperatures. According to the creep behaviors of HESA shown in Fig. 5, no obvious primary creep is observed, demonstrating that the dislocation shearing γ′ mechanism should not be applicable. Instead, thermally activated by-passing of dislocations around γ′ precipitates should be the deformation mechanism with such low applied stress;^49,50^ the inelastic deformation can essentially concentrate within the γ regions, where dislocations move by climb and glide processes to pass by γ′ particles^51–53^. Therefore, the possible sluggish diffusion of the higher entropy γ matrix and the un-rafted γ′ microstructure can contribute to lower creep rate. Furthermore, the lower stacking fault energy (SFE) of the γ matrix and higher activation energy of creep in HESA system would also be important strengthening factors. The correlation between SFE, creep activation energy and creep strain rate is described as: $\dot{\varepsilon }=A{\rm{^{\prime} }}{({\gamma }_{SF}/Gb)}^{3}{(\sigma /G)}^{5}\exp (-{\rm{Q}}/{\rm{R}}{\rm{T}})$, where A′ is a constant, γ_*SF*_ is the stacking fault energy, *G* is the shear modulus, *b* is the Burgers vector, Q is the activation energy of creep and *σ* is the applied stress^29^, so the lower SFE and higher Q of HESA can lead to lower creep rates. For materials possessing lower SFE, partial dislocations are more energetically favorable, and the spacing between partial dislocations can be increased. As a result, the dislocations bowing around γ′ particles would become more difficult, which effectively decreases the creep strain rate.

In summary, owing to higher volume fractions and higher APB energy of γ′ precipitates, HESA can show the superior tensile yield strength to those of the reported high entropy alloys from room to elevated temperatures. In addition, the high temperature strength of HESA can approach that of the Ni-based superalloy CMSX-2, and the optimizations in larger γ′ size, higher degree of misfit value may contribute to further strengthening. Regarding to creep resistances, HESA can be strengthened by the lower stacking fault energy, higher creep activation energy and stable γ′ phases; the sluggish diffusion of the high entropy γ matrix may have attributed to the higher creep activation energy. At higher temperature, although the P-raft γ′ at 982 °C has led to a more rapid increase in creep strain rate, the creep life of HESA can be compared to those of some conventional superalloys and with less refractory contents. Therefore, HESA can be a promising new type of high temperature alloy with improved cost-performance. For the future alloy designs, the minor addition of γ partitioning elements like Mo and Re would lead to the desirable negative lattice misfit for N-type rafting of γ′ and also provide solid-solution strengthening to the γ matrix^54^.

## Methods

The nominal composition of the investigated HESA is listed in Table 1. The alloy is rich in Ni, Co, Fe with a mixing entropy of the alloy to 1.60 R, which can be categorized as a high entropy alloy^14^. The term of mixing entropy is calculated by: ΔS_mix_ = −R(X_A_lnX_A_ + X_B_lnX_B_ + …), where R is the gas constant, X_A_ means the molar fraction of constituent A in whole alloy, X_B_ means the molar fraction of constituent B, and so on. Al, Ti and Nb are the γ′ forming elements for precipitation strengthening. Cr is added to provide oxidation and corrosion resistance. The slight carbon content is for grain-boundary pinning purpose, and minor amounts of Mo and W are added for solid-solution strengthening. The design of HESA was also assisted by CALPHAD-based simulation (JMatPro^55^, Ni-alloys database), and the aim is to contain more than 60 vol% of strengthening L1_2_ γ′ as commercial Ni-based superalloys. Furthermore, since the addition of refractory elements can easily promote the formation of detrimental topological-close packed (TCP) phases, the fraction of TCP was designed to be less than 1 vol% while adjusting the refractory additions. The ingot of HESA was prepared by vacuum-arc-melting process followed by directional-solidification (DS) to produce columnar microstructure; the setup of DS casting furnace has been described in our previous work^15^. The solution-heat-treatment (SHT) was then conducted at 1210 °C for 10 h to homogenize chemical segregations, and followed by a primary aging at 1000 °C for 3 h and a secondary aging at 800 °C for 20 h to grow and refine the morphology of γ′ precipitates.

A Pyris Diamond TG/DTA was used to determine the γ′ solvus temperature of HESA; 30 mg sample was put in an Al_2_O_3_ crucible with a heating rate of 10 °C/min to 1350 °C and with 400 sccm of Ar flow. A scanning electron microscope (SEM, Hitachi SU-8010) equipped with energy dispersive X-ray spectrometer (EDS) and a transmission electron microscope (TEM, Tecnai F30) were used to observe the microstructures. TEM foils were prepared by a twin-jet polisher with a solution of 30 ml perchloric acid (HClO_4_) with 300 ml methanol (CH_3_OH) and 175 ml butanol (C_4_H_9_OH) at −10 °C. An X-ray diffractometer (Shimazu XRD-6000) with a heating stage and Cu-target radiation at 30 kV/20 mA was used to obtain the characteristic γ/γ′ peaks under a vacuum environment (1 × 10^−4^ atm). The XRD specimens were in a plate form with dimension of 1 cm х 1 cm х 3 mm, and scanned at 2θ angle from 20 to 100° by 2 degree/min at room temperature and 982 °C. MDI Jade software was used to deconvolute the {200} lattice reflection and determine the peak positions of γ and γ′ for calculating the lattice misfit (δ). A strain aging method was conducted to assist measuring the compositions of γ and γ′ phase; samples were subjected to indentation after the solution-heat-treatment process stated above, and then followed by an annealing at 900 °C for 50 h. Therefore, cellular growth of γ′ would occur^20^, and resulted in the coarsened γ/γ′ morphology. In addition, the measured compositions of γ and γ′ were verified with the lever rule analyses. The diagram of ${{\rm{C}}}_{\gamma {\rm{^{\prime} }}}$ - C_γ_ vs. C_m_ - C_γ_ would be plotted, where C_γ_, ${{\rm{C}}}_{\gamma {\rm{^{\prime} }}}$ and C_m_ are the chemical compositions measured from γ, γ′ and bulk alloy, respectively; the linear fitting slope of ${{\rm{C}}}_{\gamma {\rm{^{\prime} }}}$ - C_γ_ vs. C_m_ - C_γ_ diagram should correlate with the volume fraction of γ′ phase.

Tensile tests were conducted by a MTS 810 testing machine; flat tensile specimens were prepared by electrical discharge machining. The total length of a specimen is 43 mm, while the gauge length, width, and thickness are 19 mm, 10 mm, and 1.5 mm, respectively. The specimens were tensile tested at a stain rate of 10^−3^ sec^−1^ from room temperature to 950 °C. Creep tests were performed at 750, 850 and 982 °C with a constant stress of 159 MPa using an ATS Series 2330 lever arm creep tester. The dimension of rod specimens was machined to a diameter of 6.35 mm with a gage length of 25.4 mm according to the Japanese Industrial Standard (JIS-Z-2271^56^).
